# Brief Report: Case Comparison of Therapy With the Histone Deacetylase Inhibitor Vorinostat in a Neonatal Calf Model of Pulmonary Hypertension

**DOI:** 10.3389/fphys.2021.712583

**Published:** 2021-09-06

**Authors:** Tanya J. Applegate, Greta M. Krafsur, June A. Boon, Hui Zhang, Min Li, Timothy N. Holt, S. Kelly Ambler, Benjamin A. Abrams, Daniel L. Gustafson, Karsten Bartels, Franklyn B. Garry, Kurt R. Stenmark, R. Dale Brown

**Affiliations:** ^1^Department of Clinical Sciences, Colorado State University College of Veterinary Medicine and Biomedical Sciences, Fort Collins, CO, United States; ^2^Division of Clinical Research, Medicine and Pathobiologic Services, RTI, L.L.C., Brookings, SD, United States; ^3^Departments of Pediatrics and Medicine, University of Colorado Denver, Aurora, CO, United States; ^4^Department of Anesthesiology, University of Colorado Denver, Aurora, CO, United States

**Keywords:** pulmonary hypertension, epigenetics, histone deacetylases, vorinostat, echocardiography, bovine

## Abstract

Pulmonary hypertension (PH) is an incurable condition in humans; driven by pulmonary vascular remodeling partially mediated by epigenetic mechanisms; and leading to right ventricular hypertrophy, failure, and death. We hypothesized that targeting chromatin-modifying histone deacetylases may provide benefit. In this Brief Report we describe case comparison studies using the histone deacetylase inhibitor vorinostat (suberanilohydroxamic acid, 5 mg/kg/day for the first 5 study days) in an established model of severe neonatal bovine PH induced by 14 days of environmental hypoxia. Echocardiographic, hemodynamic, and pharmacokinetic data were obtained in hypoxia-exposed (one each, vorinostat-treated vs. untreated) and normoxic vorinostat-treated control animals (*n* = 2). Echocardiography detected PH changes by day 4 and severe PH over 14 days of continued hypoxic exposure. RV dysfunction at day 4 was less severe in vorinostat-treated compared to untreated hypoxic calves. Cardioprotective effects were partially maintained following cessation of treatment through the duration of hypoxic exposure, accompanied by hemodynamic evidence suggestive of reduced pulmonary vascular stiffening, and modulated expression of HDAC1 protein and genes involved in RV and pulmonary vascular remodeling and pathological RV hypertrophy. Control calves did not develop PH, nor show adverse cardiac or clinical effects. These results provide novel translation of epigenetic-directed therapy to a large animal severe PH model that recapitulates important features of human disease.

## Introduction

Pulmonary hypertension (PH) is a progressive, incurable disease resulting in significant health and economic burdens (Hyduk et al., 2005; Sikirica et al., 2014). Chronic PH leads to right ventricular (RV) hypertrophy and ultimately RV failure and death. Five groups of PH are recognized, with groups 2 (PH due to left heart disease) and 3 (PH due to chronic lung disease or hypoxia), alone or in combination, by far the most common and lethal forms (Simonneau et al., 2009, 2013; Wijeratne et al., 2018). Nearly a quarter of all individuals with elevated pulmonary arterial pressures (PAP) fall into group 3 classification (Strange et al., 2012), estimated at > 500,000 individuals in the US (Pugliese et al., 2015; Cuttica, 2016; Klinger, 2016). There is a lack of drug therapies specifically targeting and approved for use in this patient group. Moreover, currently approved PH vasodilator therapies do not exert direct cardioprotective actions.

The causes of PH are multifactorial and involve the loss of peripheral pulmonary vessels, chronic or recurrent hypoxia, and altered expression of vascular and inflammatory mediators. Common pathophysiologic characteristics of PH include excessive vasoconstriction and vascular remodeling involving all layers of the vessel (adventitia, media, and intima) throughout the pulmonary vascular tree, resulting in significant increase in RV afterload, and the sequelae of RV dysfunction (Schermuly et al., 2011).

Importantly, perivascular and adventitial expansion that continues despite currently approved vasodilator therapies is consistently associated with pulmonary arterial specimens obtained from biopsy or lung transplant patients (Stacher et al., 2012; Ghigna et al., 2017). Adventitial expansion reflects activation of resident fibroblasts and macrophages, as well as recruitment of circulating monocytes and mesenchymal cells (Davie et al., 2004; Frid et al., 2006). This cellular activation leads to early and persistent perivascular inflammation with appearance of chemokines and associated receptors (Burke et al., 2009). Studies from our laboratories have shown that this expansion reflects activation of pro-inflammatory, proliferative, anti-apoptotic, and metabolic gene programs by resident fibroblasts (PH-Fibs). PH-Fibs in turn promote inflammatory activation, migration, and adhesion of target macrophages. The durable phenotype of activated PH-Fibs is regulated by an epigenetic mechanism mediated through histone deacetylases suggesting that epigenetic mechanisms may perpetuate the inflammatory perivascular remodeling in chronic PH (Li et al., 2011; Wang et al., 2014; Zhang et al., 2017). Epigenetic activation of additional cell types important in PH, especially vascular smooth muscle cells, and their reversal by HDAC inhibitors has also been reported (Stratton and McKinsey, 2015).

Epigenetic control of gene expression can arise from DNA methylation, histone modification or gene silencing by microRNA (Pugliese et al., 2015). Changes in chromatin geometry are induced by acetylation and deacetylation of histones by histone acetyl transferases (HAT) or histone deacetylases (HDAC) (Wagner et al., 2013; Eom and Kook, 2014; Tao et al., 2014). Both HAT and HDAC have post-translational actions on many other proteins and thus have broad potential for impacting cell function (Tao et al., 2014). There are 18 isoforms of HDAC molecules, divided within four classes. The functions of HAT and HDAC are influenced by inhibitors and activators. HDAC inhibitors have been shown to have anti-inflammatory effects in animal models of inflammatory diseases, fibrotic vascular disease, and cancer. Clinical trials of the Class 1 HDAC inhibitors have been reviewed (Ververis et al., 2013).

Targeting epigenetic mechanisms mediated by histone acetylation offers a novel approach to PH therapy (Stratton and McKinsey, 2015). Although an initial study using a non-selective HDAC inhibitor in a rat model reported adverse cardiac effects, more recent studies *in vitro* and in rodent models using class-selective HDAC inhibitors have shown promise (Bogaard et al., 2011; Chelladurai et al., 2021). We showed elevated class I HDACs-1,2, and 3 expression and activities in bovine PH-fibs. Treatment with the class I/II HDAC inhibitors vorinostat or apicidin reversed the activated fibroblast phenotype (Li et al., 2011). Zhao et al. (2012) showed elevated HDAC-1 and 5 expression in human PAH lung; treatment with the Class I inhibitor valproic acid inhibited hypoxia-induced PH in rats. In rat models of hypoxia-induced pulmonary hypertension, treatment with Class I HDAC inhibitors MCD-0103 or MS-275 preserved RV function and attenuated RV hypertrophic and pro-fibrotic remodeling; and valproic acid attenuated RV hypertrophy (Cho et al., 2010; Cavasin et al., 2012). Accumulating evidence suggests HDAC inhibitors may also be of benefit in left heart disease (McKinsey, 2012).

Limitations of rodent models in PH make it essential to extend the studies to larger animals (Stenmark et al., 2009). More similar to humans and in contrast to rodents, calves exposed to 14 days of environmental hypoxia develop severe PH, resulting in structural remodeling and profound molecular re-programming within the pulmonary vasculature, accompanied by evidence of RV dysfunction and hypertrophic remodeling (Stenmark et al., 1987; Lemler et al., 2000; Bartels et al., 2016). To test the hypothesis that therapy targeting epigenetic regulation would attenuate RV dysfunction in response to PH, we performed case comparison studies to evaluate the histone deacetylase inhibitor vorinostat (5 mg/kg/day) in a well-established neonatal calf model of hypoxia-induced PH. We utilized echocardiography to non-invasively and semi-quantitatively assess early cardiovascular responses to PH, and potential therapeutic benefits of vorinostat. We measured cardiopulmonary hemodynamics, molecular correlates of cardiac and pulmonary remodeling by qPCR, and analyzed pharmacokinetics of vorinostat administration.

## Materials and Methods

### Experimental Animals and Study Design

Animal procedures were approved by the Colorado State University IACUC. Study procedures were performed on awake calves under manual restraint in lateral recumbency.

The bovine calf hypoxia model of PH previously reported (Stenmark et al., 1987; Lemler et al., 2000; Bartels et al., 2016) consistently produces significant pulmonary hypertension (mean PAP > 100 mmHg) and subsequent tissue changes on post-mortem examination (Stenmark et al., 1987). Animals display blood gas PaO_2_ and PaCO_2_ values consistent to severe hypoxia and PH (Stenmark et al., 1987; Lemler et al., 2000). Male Holstein dairy calves at 7 days of age (45–50 kg) were sourced from a local dairy farm. This study utilized a case comparison design. Calves were assigned to the following treatments: (1) Hypoxic, vorinostat administration (one calf); (2) Hypoxic, no treatment (one calf); (3) Normoxic, vorinostat administration (Control, two calves). Hypoxic calves (Groups 1 and 2) were exposed to hypobaric hypoxia at simulated elevation of 10,000 feet for 48–72 h to allow acclimation then exposed to hypobaric hypoxia at the study condition of 15,000 feet simulated elevation (P_*B*_ = 430 mmHg, Hypo-Hyperbaric Chamber Facility, Colorado State University Department of Physiology). The standard exposure interval was 14 days at this elevation. Control normoxic calves remained at ambient Fort Collins pressure (4,950 feet P_*B*_ = 630 mmHg).

Vorinostat (Selleckchem, Houston, TX, United States) was capsulated with microcrystalline cellulose as excipient. Vorinostat (200–250 mg/d) was administered as a single oral dose every 24 h beginning on the first day of hypoxia induction at 15,000 feet in Group 1, and the equivalent day for Group 3, and continued for 5 days. Vorinostat dosage was 180 mg/m^2^ body surface area/day determined according to the conversion BSA(m^2^) = 0.14 × Body Wt (kg)exp(0.57) (Berman, 2003), 235 mg for a 50-kg calf, equivalent to 4.7 mg/kg/d. This dosing regimen was selected to minimize adverse effects based on guidelines for human pediatric patients (Lia Gore MD, Center for Cancer and Blood Disorders, Children’s Hospital Colorado, personal communication.) Group 2 calves received no drug treatment. At this age, the calf digestive system has not matured and the abomasum functions as the sole stomach similar to humans. No adverse clinical signs attributable to vorinostat administration were observed either during or following active dosing in animals treated in normoxic or hypoxic conditions. All standard blood chemistry values remained within normal reference intervals (data not shown).

### Echocardiography

Echocardiography was utilized for non-invasive sequential assessment of cardiopulmonary function as described previously (Lemler et al., 2000; Bartels et al., 2016). Studies were obtained from two normoxic, vorinostat administered calves and two hypoxic calves, one vorinostat treated and one untreated. The progression of pulmonary hypertension was evaluated by sequential echocardiography at the study condition (15,000 feet or normoxia) on study days 4, 8 and terminal day 14. Parasternal long and short axis recordings were obtained over 3–4 cardiac cycles, together with M-mode traces as indicated, using GE Echo PACS (General Electric, Chicago, IL, United States) or MicroDicom^1^ software. Echocardiographic findings consistent with PH (abnormal RV size and function), or interventricular septum abnormalities including abnormal motion or flattening were evaluated (Boon, 2011). Septal motion and flattening were further quantitated by eccentricity index EI on short-axis views with EchoPACS. Tricuspid or mitral valve regurgitation was scored qualitatively as trace, mild, moderate or severe. Global RV function was assessed qualitatively. When RV images on long-axis included the entire chamber, RV fractional area change (FAC) was calculated with EchoPACS. RV fractional shortening was measured from M-mode recordings using MicroDicom. Calves were monitored following each non-terminal echo procedure for recovery including standing and interacting normally, and normal milk intake.

### Hemodynamics

Standard measurements of PAP and cardiac output were performed by right heart catheterization concurrent with the final echo in both the two normoxic and two hypoxic calves on study day 14 as described previously (Stenmark et al., 1987; Lemler et al., 2000).

### Pharmacokinetics

Repeated blood samples were obtained at specified intervals from catheters placed in the jugular vein of four normoxic vorinostat treated calves for assessment of pharmacokinetics over the initial 24 h of dosing. Vorinostat and its metabolites were quantitated in serum by mass spectroscopy at the Colorado State University-University of Colorado Cancer Center Pharmacology Core Laboratory. Pharmacokinetic parameters were calculated by a non-compartmental model using Phoenix WinNonlin v8.1 (Wagner, 1993).

### Tissue Sampling and Quantitative Real-Time PCR

At the conclusion of the study period, calves were deeply anesthetized and euthanized. Tissue samples from right ventricle free wall and distal lung parenchyma were snap frozen in liquid nitrogen for laboratory analysis. RNA isolation and quantitative PCR were performed as described previously (Frid et al., 2009). mRNA abundance was quantitated relative to HPRT by the delta Ct method. The following primers were used (5′–3′). Fibronectin, Extracellular Domain A (ED-A Fn), Fwd:ACCTACTGGAGCCCTGAGGA, Rev:CGTGCAAGGCAACCACACTG; Tenascin C (TnC), Fwd:G ATCTGAGCCCATCCACCTA, Rev:GGAATCGGTACAGGAC TCCA; Osteopontin (SPPA), Fwd:CAGAGTCCAGATGC CACAGA, Rev:GGAAAGCTCGCTACTGTTGG; Periostin (POSTN), Fwd:GCCACTTTGTCTCCCATGAT, Rev:CAC TCTTTGCTCCCACCAAT; Skeletal muscle alpha-actin (ACTA1), Fwd:ATCGCCGACCGCATGCAGAA, Rev:GAAGGT GGACAGCGAACGCA; Brain natriuretic peptide (NPPB), Fwd:ATGCGCGACTCTGGCTGCTT, Rev:TGCAGCCAGGAC CTCTTCTT; Hypoxanthine ribosyl transferase (HPRT), Fwd:CTGGCTCGAGATGTGATGAA, Rev:CAACAGGTCGGC AAAGAACT.

### Immunoblotting

Procedures for immunoblotting and densitometry were performed as described previously (Li et al., 2011). HDAC antibodies were obtained from Cell Signaling Technology (HDAC1, #5356; HDAC5, #20458).

## Results

### Echocardiography

**FIGURE 1:**
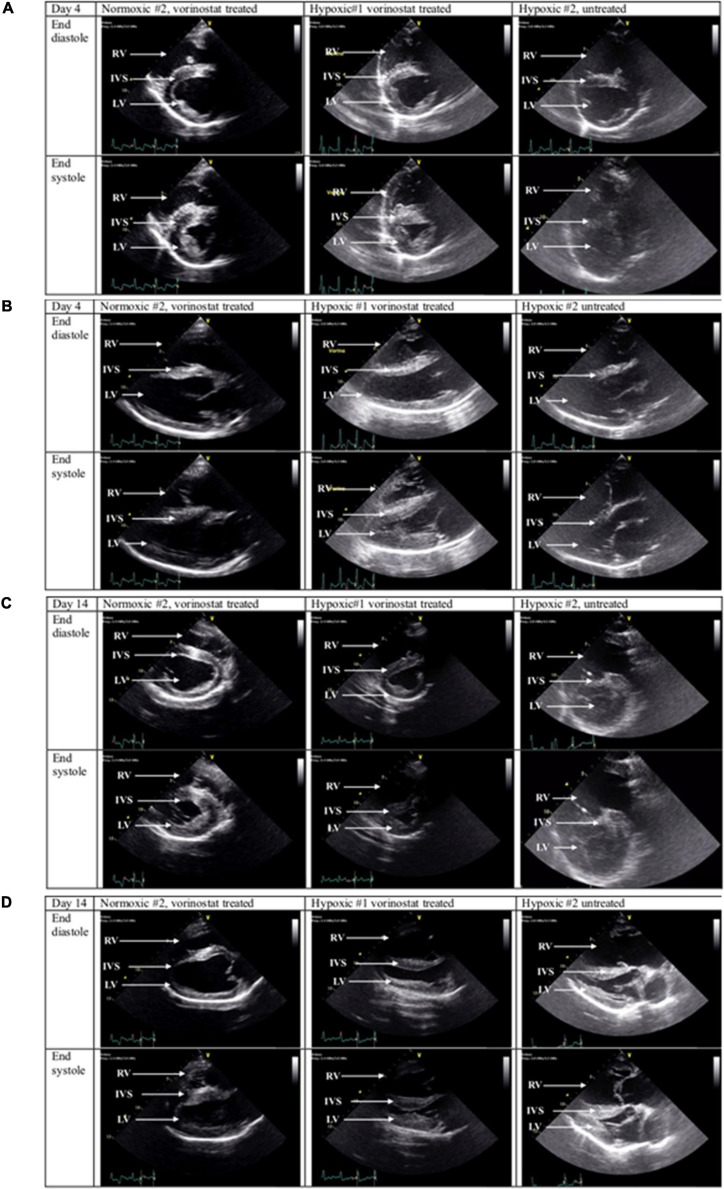
Echocardiographic imaging of vorinostat effects in calves with hypoxia-induced pulmonary hypertension. Calf environmental exposures, vorinostat treatments, and echocardiography were performed as described in section “Materials and Methods.” Representative echo images captured at systole and diastole are shown for one normoxic vorinostat-treated calf, one hypoxic vorinostat-treated calf, and one hypoxic untreated calf. Note: Representative echo recordings of cinematic loops are shown in Supplementary Videos 1, 2. **(A)** Experimental day 4, parasternal short axis views. **(B)** Experimental day 4, parasternal long axis views. **(C)** Experimental day 14, parasternal short axis views. **(D)** Experimental day 14, parasternal long axis views.

**FIGURE 2:**
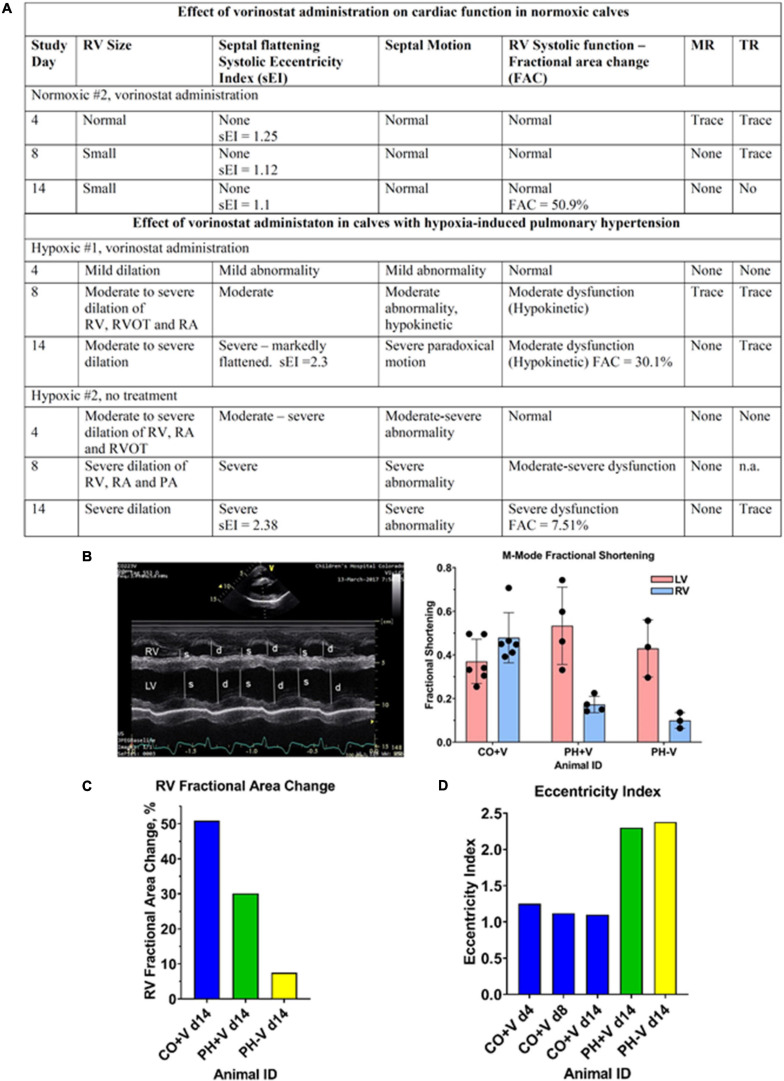
Summary of qualitative and quantitative echo assessments. Echo records were independently evaluated by qualified sonographers as described in section “Materials and Methods.” **(A)** Summary of qualitative echo findings. **(B)** Quantitation of RV and LV fractional shortening. Left panel, representative M-mode tracing, showing RV and LV chamber dimensions at systole (s) and diastole (d). Right panel, quantitation of RV fractional shortening. Each dot represents the average of fractional shortening values determined over 3–4 cardiac cycles in a single M-mode trace; multiple traces were obtained for each condition. **(C)** Quantitation of RV Fractional Area Change (FAC). FAC was determined from cinematic echo recordings over multiple cardiac cycles for each condition. **(D)** Quantitation of Eccentricity Index (EI). EI was determined from cinematic echo recordings over multiple cardiac cycles for each condition.

Marked echocardiographic changes were found in calves with hypoxia-induced PH. Both untreated and vorinostat-treated hypoxic calves demonstrated RV dilation with decreased systolic emptying as early as day 4. Representative short and long axis images for one calf each, normoxic-vorinostat, hypoxic-vorinostat, and hypoxic-no vorinostat, obtained at d4, are shown in Figures 1A,B, respectively. By day 14, both hypoxic calves demonstrated severe septal flattening, RV dilation, and decreased emptying. However, the degree of RV dysfunction appeared consistently less severe in the vorinostat-treated hypoxic calf than the untreated calf throughout days 4, 8, and 14. Although RV dysfunction was present in both hypoxic calves by day 14, the dilation and decreased systolic emptying qualitatively appeared worse in the untreated hypoxic calf. Corresponding images at day 14 are shown in Figures 1C,D. Figure 2A summarizes echo findings from these animals, representing independent assessments from individuals experienced with large animal (TJA, JAB), human (BAA), or rodent (SKA) echo. Echo images shown in Supplementary Figure 1 corroborate these findings. PH causes RV expansion and septal distortion in the untreated calf whereas vorinostat treatment reduced septal distortion. Further comparisons are shown in video records from control, hypoxic-treated, and hypoxic-untreated calves from studies on days 4 and 14 (Supplementary Videos 1, 2, SAX Comparisons, PLAX Comparisons, respectively). Collectively these observations suggest that early and limited duration treatment with vorinostat exerts modest but appreciable sustained cardioprotective actions in response to hypoxia-induced pulmonary hypertension.

In addition to qualitative evaluations described in Figures 1, 2A and Supplementary Figures 1, 2, the cardioprotective effects of vorinostat therapy were confirmed by quantitation of parameters of RV performance. Figure 2B shows fractional shortening determined from M-mode images as a surrogate measurement of ejection fraction. RV fractional shortening on day 14 was 50% in the control calf compared to 20% in the vorinostat-treated PH calf vs. 12% in untreated PH. As expected, LV fractional shortening (∼50%) was unaffected by PH. Similarly, RV Fractional Area Change (FAC), a measure of RV ejection fraction, was 50.9% in the control calf on day 14, whereas RV FAC on day 14 was 30.1% in the vorinostat-treated hypoxic calf compared to 7.51% in the untreated hypoxic calf (Figure 2C). Together, these two parameters show that vorinostat confers lasting protection of RV contractile function in PH.

The cardioprotective actions of vorinostat occurred independently of reducing PH pressure overload. Figure 2D shows echo measurements of eccentricity index (EI), reflecting septal flattening induced by pressure overload. The EI for this normoxic calf was 1.25, 1.11, and 1.1 on days 4, 8, and 14 respectively. On day 14, the vorinostat-treated and un-treated hypoxic calves had similar EI values (2.3 and 2.38, respectively), substantially increased from normoxic controls, and reflecting equivalent pressure overload.

Vorinostat treatment in control normoxic calves was not associated with echocardiographic evidence of negative impacts on cardiac function. As shown in Figures 1, 2, a normoxic calf treated with vorinostat had normal RV size and function in all echocardiography examinations. Representative echo images obtained on day 4 (Figures 1A,B) and day 14 (Figures 1C,D) showed no changes in RV diastolic volume or systolic emptying. The second normoxic calf showed equivalent results (data not shown).

### Hemodynamics

As shown in Supplementary Table 1, hemodynamic measurements of calves in the hypoxic groups demonstrated robust increases in mean and systolic PA pressures compared to normoxic controls, in agreement with our published reports on this animal model (Stenmark et al., 1987; Lemler et al., 2000). Although the vorinostat-treated and untreated PH calves had similar mean PA pressures, the vorinostat-treated calf tended toward reduced PA systolic and pulse pressures, and increased cardiac output compared to the untreated calf, again suggesting modest protection of function. Vorinostat treatment did not affect hemodynamic parameters in the normoxic calves compared to our previous studies.

### Molecular Correlates of Vorinostat Actions

**FIGURE 3:**
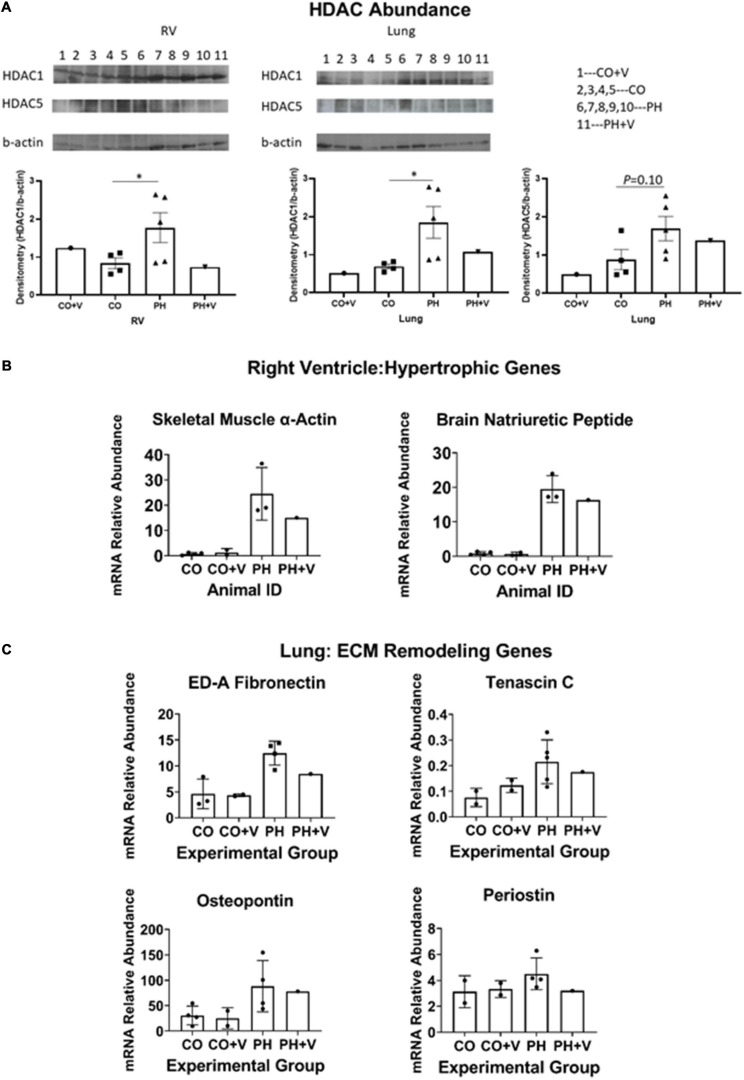
Molecular correlates of vorinostat treatment. **(A)** Immunoblotting HDAC abundance. RV and lung homogenates were prepared and HDAC abundance was determined by immunoblotting as described in section “Materials and Methods.” Additional untreated control and hypoxic animals were analyzed in parallel for comparison. Protein abundance was quantitated densitometrically relative to beta-actin. *p < 0.05 vs. control (unpaired two-tailed *t*-test). **(B)** Representative genes in RV hypertrophic remodeling. Abundances of indicated mRNAs were quantitated by real-time PCR as described in section “Materials and Methods.” Animals used in the present study were analyzed together with samples from additional untreated hypoxic and normoxic calves (*n* = 4 each) that were managed according to the same animal protocols described in section “Materials and Methods,” and studied around the same period of time. **(C)** Representative genes in pulmonary remodeling. Determinations were performed as described for **(A)** and in section “Materials and Methods.”

We quantitated the protective actions of vorinostat treatment on HDAC abundance by immunoblotting. Based on literature reports we evaluated HDAC1 as a representative class I HDAC and HDAC5 as class IIa. For purpose of quantitative comparison, results from the animals used in the present study were analyzed together with samples from additional untreated hypoxic and normoxic calves (*n* = 3 each) that were managed according to the same animal protocols described in section “Materials and Methods.” Figure 3A shows that PH significantly upregulates expression of HDAC1 in lung homogenates, consistent with our previous results with adventitial fibroblasts, and moreover elevates HDAC1 expression in RV. Vorinostat treatment over the initial 5 days of hypoxia tended to decrease expression of HDAC1 in both lung and RV, which was still detectable at the end of 14 days hypoxic exposure. HDAC5 expression in lung shows similar qualitative trends but the abundance was much lower and did not reach statistical significance. There was insufficient HDAC5 in RV for interpretation.

We next evaluated expression of target gene mRNAs involved in RV and pulmonary vascular remodeling using real-time PCR, as shown in Figures 3B,C. Figure 3B shows RV expression of brain natriuretic peptide (BNP) and skeletal muscle alpha-actin, characteristic mRNAs indicating pathologic hypertrophic RV remodeling that were previously shown to be attenuated by HDAC inhibitor treatment in rat hypoxic PH (Cavasin et al., 2012). Vorinostat treatment tended to decrease expression of both mRNAs compared to calves with untreated PH. mRNA abundance of control calves was not affected by vorinostat treatment. In additional experiments, we performed pairwise qPCR comparisons of the hypoxic vorinostat-treated vs. –untreated calves and showed that vorinostat treatment also reduced expression of a number of pro-inflammatory mRNAs including IL-6, CSF2, SDF1, IL1β, IL-1RA, TLR2, and the matrikine POSN (Supplementary Figure 2). Similarly, Figure 3C shows expression of genes involved in initiating lung vascular ECM remodeling: the structural proteins fibronectin-extracellular domain A (ED-A Fn) and tenascin C (TNC); and the matrikines osteopontin and periostin. We previously showed these mRNAs are upregulated in pulmonary vessels and cultured adventitial fibroblasts from PH animals (Burke et al., 2009; Li et al., 2011). Vorinostat treatment tended to reduce expression of these mRNAs in hypertensive calves compared to untreated hypertensive calves. Abundance of the corresponding mRNAs in normoxic animals was not affected by vorinostat treatment. Together these results suggest vorinostat treatment modulates pulmonary vascular remodeling, together with beneficial effects on hypertrophic reprogramming of gene expression in the RV. These effects are at least partially maintained following the cessation of therapy.

### Pharmacokinetics

**FIGURE 4:**
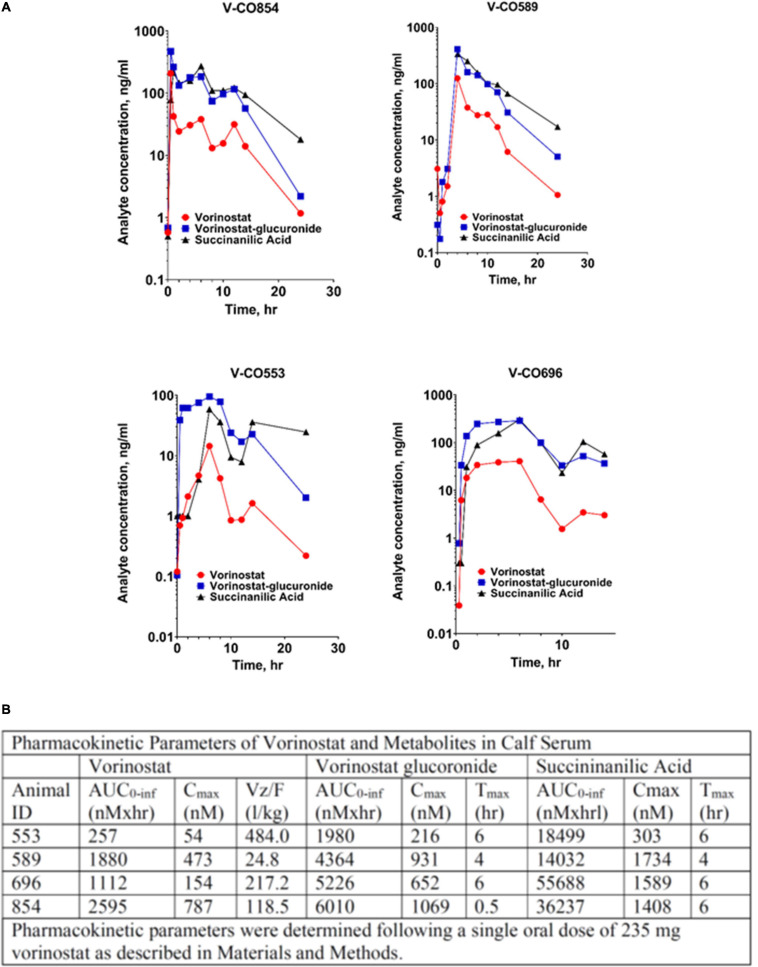
Pharmacokinetics of vorinostat administration and metabolism in calves. **(A)** Serum concentrations of vorinostat and metabolites following oral dosing. Vorinostat (235 mg) was administered orally. Blood was drawn at indicated intervals and analyzed for vorinostat and its metabolites as described in section “Materials and Methods.” Data from four replicate animals are shown. **(B)** Pharmacokinetic parameters of vorinostat and metabolites. Pharmacokinetic parameters were calculated from the time course data in A as described in section “Materials and Methods.” AUC, area under the curve, integrated sum of total amount of analyte; Cmax, maximum serum concentration; Tmax, time of maximum serum concentration; Vz/F, volume of distribution normalized to oral bioavailability.

Serum concentrations of vorinostat and its principal metabolites, the O-glucuronide conjugate and succinanilinic acid, the product of β-oxidation, following single dose oral administration are shown from four normoxic control calves in Figure 4A. Pharmacokinetic parameters calculated from these data are shown in Figure 4B. Vorinostat rises to peak serum concentrations of ∼400 nM within 4 h, with an integrated area under the concentration × time curve ∼1,500 nM•h. Serum vorinostat returns to undetectable levels within 24 h. The composite parameter of apparent volume of distribution relative to oral bioavailability, Vz/F, was estimated at 217 L/kg for a single oral dose of 235 mg vorinostat and 50 kg calf body weight. It was not feasible to determine bioavailability or clearance rates from these data. Vorinostat administration is accompanied by the near-simultaneous appearance of its metabolites, in quantitative excess of the parent drug. This pattern was consistent in the four animals, although quantitative variability in the pharmacokinetic profiles was observed.

## Discussion

In this Brief Report, we show for the first time in a large animal model of severe PH, cardioprotective benefit of therapy with vorinostat, an epigenetic-targeted histone deacetylase inhibitor. This study provides a template for further investigation with additional epigenetic-targeted agents either alone or in combination with standard therapies in large animal models of PH.

Echocardiography to evaluate cardiac changes has been shown previously to be useful in the calf model for hypoxia-induced PH (Lemler et al., 2000), including quantitative evaluation of RV strain parameters to reflect worsening RV function (Bartels et al., 2016). These studies focused on endpoint assessment of PH following hypoxic exposure, whereas information on development of PH during the interval of hypoxic exposure is limited. The present study demonstrates the utility of serial non-invasive echocardiography to assess the onset and progression of PH and RV dysfunction in response to chronic hypoxia. Echocardiography demonstrated signs of RV pressure overload as early as 4 days of hypoxia. Our findings of RV dilation and septal flattening by 4 days of hypoxia are consistent with observations from human neonates that RV dilation and septal flattening are the most reliable early predictors of adverse pulmonary vascular disease outcomes (Mourani et al., 2008, 2015). Hypoxic calves developed severe RV dilation and dysfunction by study day 14.

We observed modest but self-consistent qualitative echo results indicating cardioprotective effects of vorinostat during the 5 days dosing interval, that were at least partially maintained throughout the duration of hypoxic exposure as verified by quantitation of Fractional Area Change and Fractional Shortening. These beneficial actions of vorinostat were present despite substantial hypoxia-induced PH in the treated and untreated animals as measured echocardiographically by Eccentricity Index and confirmed by right heart catheterization. We did not have normoxic, untreated calves to compare to these calves, but comparable echocardiographic findings using similar protocols for normoxic untreated calves were described in our previous report (Bartels et al., 2016). Alterations in cardiac rhythm including QTc prolongation, have been reported with HDAC inhibitors (Iwamoto et al., 2013). We observed no evidence for adverse cardiac effects based on echo and hemodynamic criteria.

Bovine hypoxia-induced PH resulting in right heart failure was described over one hundred years ago in cattle living at high altitude (Rhodes, 2005; Drexler et al., 2008; Burke et al., 2009). PH in cattle shows similar progression and variability in outcomes to human PH, thus providing a natural model of disease (Rhodes, 2005; Newman et al., 2011; Ghigna et al., 2017). Hypoxia induced PH results in structural remodeling throughout the pulmonary arterial tree, including thickening of intimal, medial, and adventitial layers of proximal and distal pulmonary arteries, proliferation of vasa vasorum, smooth muscle cell hypertrophy, and expansion and inflammatory-fibrotic remodeling of the adventitia (Stenmark et al., 2006; Ghigna et al., 2017). Vascular stiffening occurs as a consequence of these changes and exerts multiple adverse effects that are recognized as early and independent drivers of PH progression (Schafer et al., 2016). First, the loss of vascular compliance increases RV afterload independent of static PA pressure and decreases the mechanical efficiency of right ventricular-pulmonary arterial coupling; and second, stiffening increases flow pulsatility in distal arteries, inducing inflammatory gene expression, leukocyte adhesion, and cell proliferation in vascular endothelial cells (Li et al., 2009). While quantitatively modest, our data are consistent with the idea that epigenetic-directed therapy with vorinostat modulates ECM gene expression underlying increased vascular stiffness. Furthermore, our hemodynamic results show that vorinostat treatment in PH did not lower mean PA pressure yet slightly reduced systolic PA pressure and substantially reduced PA pulse pressure, while increasing cardiac output compared to the untreated hypoxic calf. Whereas mean PA pressures reflect static pulmonary vascular resistance, PA systolic and pulse pressures reflect oscillatory characteristics of pulsatile blood flow arising from proximal arterial stiffness (Schafer et al., 2016). Our data therefore suggest that vorinostat acts preferentially on processes related to vascular stiffening. In their study using hypoxic rats with sustained HDAC inhibitor dosing (21 days), Cavasin et al. reported significantly lowered mPAP with reduced PA medial hypertrophy and stiffening, together with evidence of direct beneficial actions on RV remodeling. Studies in beef cattle raised at high altitude showed that increased mean, systolic, and PA pulse pressures, independently predicted increased risk of mortality due to right heart failure (Neary et al., 2016). Taken together, we hypothesize that the beneficial actions of vorinostat on pulmonary vascular stiffening in our study lead to the observed improvements in cardiac morphology and performance, and molecular markers of pathologic remodeling. HDAC inhibitors thus target a novel and important component of PH pathophysiology: the epigenetic control of structural remodeling driving pathophysiology and progression of PH. HDAC inhibitor treatment holds the potential to prevent or reverse the vascular remodeling that underlies worsening PH.

Analysis of vorinostat pharmacokinetics complements the findings on its therapeutic actions. Pharmacokinetic data show vorinostat reached peak serum concentrations of ∼400 nM within ∼4 h, with total absorption ∼1,500 nM•h, and declined to undetectable levels by 24 h after administration. Vorinostat pharmacokinetic parameters in human subjects have been comprehensively reviewed (Iwamoto et al., 2013). By comparison to our data, vorinostat at the comparable weight-adjusted adult dose of 400 mg daily yields Cmax of 1,000–1,500 nM and AUC 4,000–6,000 nM•h. Pediatric patients, dosed equivalently to our studies, tend to exhibit higher vorinostat exposure than adults, although with greater individual variability as we observe. The time course of vorinostat appearance is similar in calves to post-fed humans, with Tmax ∼4 h. Vorinostat is largely metabolized by glucuronidation and hydrolysis followed by oxidation. The rapid appearance of these metabolites is associated with first-pass metabolism at the liver circumventing systemic absorption (Sandhu et al., 2007). Reported half-life of vorinostat in humans and other animal species is approximately 2 h (Mohamed et al., 2012; Fraczek et al., 2013; Iwamoto et al., 2013). There is relatively little accumulation of vorinostat in humans even after repeated administration, similar to our results. However, we note potentially significant differences in vorinostat metabolism. In humans, the AUC of the initial vorinostat:glucuronide conjugate is typically 3–4 times that of vorinostat and the terminal oxidation metabolite succinanilic acid is 10–13 times vorinostat. Our data show a similar ratio of vorinostat:glucuronide AUC (∼3-fold), but succinanilic acid: vorinostat was ∼20-fold, consistent with enhanced metabolic clearance in the calf. In addition, the poor aqueous solubility and lipid partition coefficient of vorinostat reduce its absorption, leading to Class IV designation in the Biopharmaceutics Classification System. Bioavailability of oral vorinostat in humans is estimated at 43% (Fraczek et al., 2013), with considerable variation across species as bioavailability is 11% in dogs and only 2% in rats (Fraczek et al., 2013). The combined low absorption and rapid first pass elimination of vorinostat are consistent with the artificially elevated apparent volume of distribution that we observe. Taken together, these results suggest that the modest cardioprotective effects in our study reflect pharmacokinetic limitations rather than lack of therapeutic efficacy. Optimization of the dosing regimen including prolonged dosing throughout the hypoxic exposure and choice of epigenetic agent(s), offers promise for improved therapeutic benefit in PH. Drugs targeting additional epigenetic regulatory mechanisms are also in development; notably, inhibitors of the bromo-domain family of chromatin “reader” proteins may offer improved efficacy and lack of potential toxicity (Meloche et al., 2015; Hu et al., 2019).

Although the present case comparison studies show promise for further work, we clearly recognize that the limited number of animals in this study are insufficient to draw quantitatively rigorous conclusions. Additional limitations to address are identified. First, improved echocardiographic imaging will be necessary to allow consistent quantitation of RV performance (TAPSE, Fractional Area Change, wall motion S wave, myocardial performance index) and pulmonary vascular stiffening (PA acceleration rate, E/A ratio, velocity-time index). Second, further work will be needed to optimize dose, administration frequency and/or choice of single or combination agents to improve therapeutic effects. It will also be important to validate the pharmacokinetic analysis under hypoxic conditions. Third, we studied calves at the onset of hypoxia-induced PH, whereas most patients present with advanced disease. Epigenetic-targeted therapies specifically address this scenario; protocols to study PH reversal will be an important approach for future research.

In conclusion, this study provides initial data on the HDAC inhibitor vorinostat as a novel epigenetically-targeted PH therapy. We demonstrate the utility of echocardiography for serial non-invasive assessment of early alterations in cardiopulmonary function, and evaluating therapeutic benefit. These results establish a framework for future translational studies in this large animal model as a bridge to improved therapies for this important and costly human disease.

## Data Availability Statement

The original contributions presented in the study are included in the article/Supplementary Material, further inquiries can be directed to the corresponding author/s.

## Ethics Statement

The animal study was reviewed and approved by the Colorado State University Institutional Animal Care and Use Committee.

## Author Contributions

TA, GK, KB, FG, KS, and RB: study design. TA, GK, JB, HZ, ML, TH, and RB: experimental procedures. TA, JB, HZ, ML, SA, BA, DG, and RB: data analysis. TA, SA, KS, and RB: manuscript writing. KS: funding. All authors reviewed and approved the final submitted manuscript.

## Conflict of Interest

GK was employed by RTI, LLC. The remaining authors declare that the research was conducted in the absence of any commercial or financial relationships that could be construed as a potential conflict of interest.

## Publisher’s Note

All claims expressed in this article are solely those of the authors and do not necessarily represent those of their affiliated organizations, or those of the publisher, the editors and the reviewers. Any product that may be evaluated in this article, or claim that may be made by its manufacturer, is not guaranteed or endorsed by the publisher.

## Footnotes

1
https://www.microdicom.com
