# Supplementary figures and images for: Brief Report: Case Comparison of Therapy With the Histone Deacetylase Inhibitor Vorinostat in a Neonatal Calf Model of Pulmonary Hypertension
Source: Front Physiol. 2021 Sep 6;12:712583. doi: 10.3389/fphys.2021.712583 (PMC8450341; doi:10.3389/fphys.2021.712583)

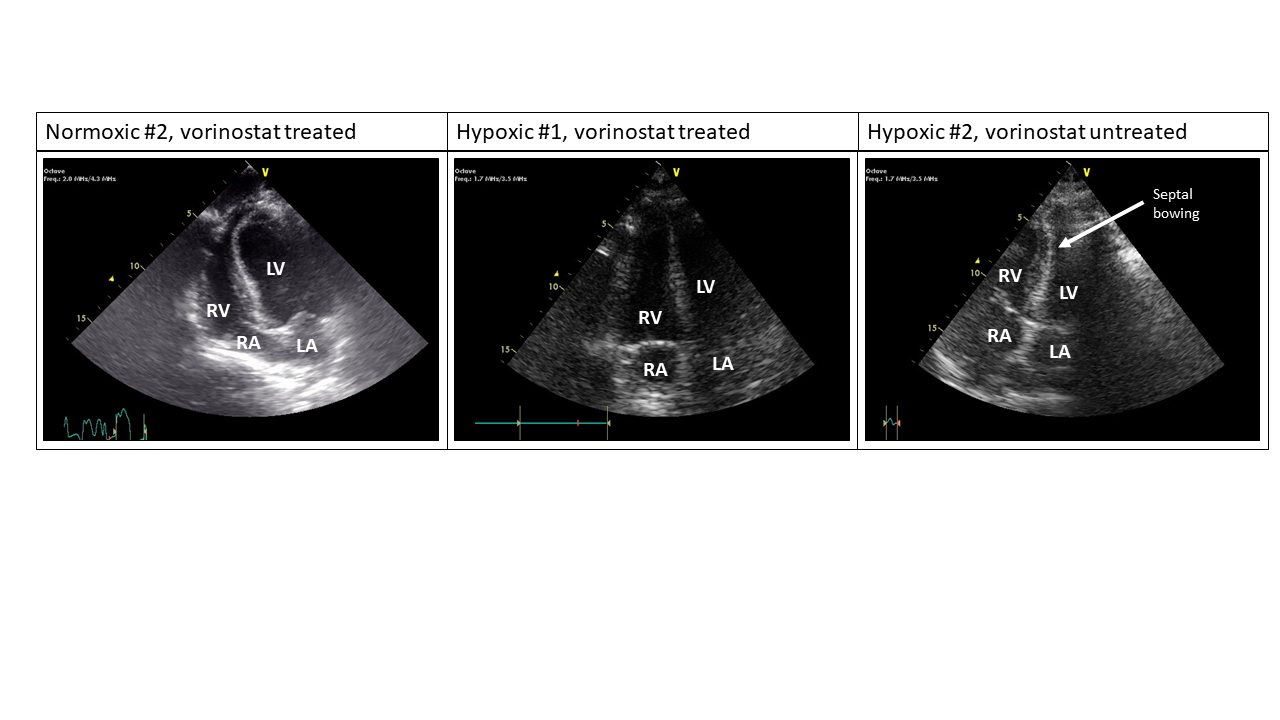

Supplement: Supplementary file 1 [file Image_1.TIF]

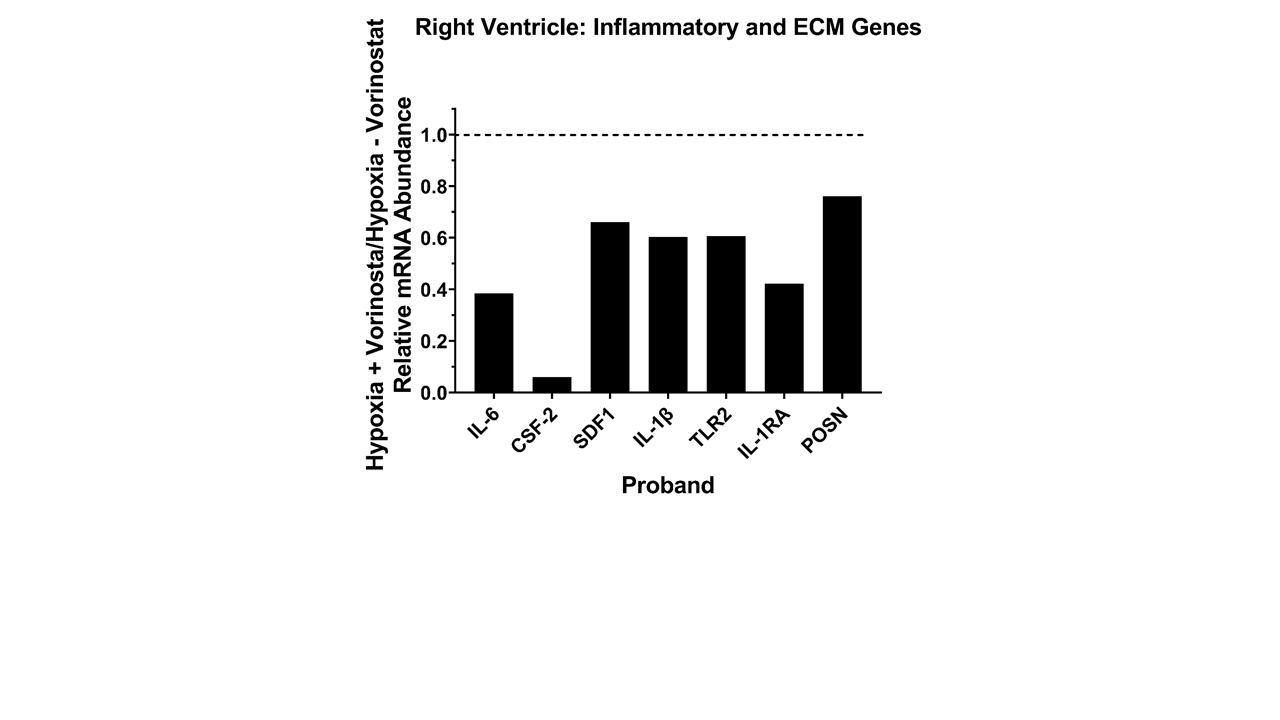

Supplement: Supplementary file 2 [file Image_2.TIF]
