# Supplementary material for: Brief Report: Case Comparison of Therapy With the Histone Deacetylase Inhibitor Vorinostat in a Neonatal Calf Model of Pulmonary Hypertension
Source: Front Physiol. 2021 Sep 6;12:712583. doi: 10.3389/fphys.2021.712583 (PMC8450341; doi:10.3389/fphys.2021.712583)
Supplement: Supplementary file 3 [file Table_1.DOCX]

**Data Supplement**

Tanya J. Applegate et al., Vorinostat therapy for PH in neonatal calves

Supplemental Table 1. Hemodynamic parameters

| Hemodynamic Parameter | Normoxic vorinostat treated #1 | Normoxic vorinostat treated #2 | Hypoxic vorinostat treated | Hypoxic untreated |
| --- | --- | --- | --- | --- |
| Mean PA Pressure | 25.1 | 19.4 | 97.6 | 90.7 |
| Systolic PA Pressure | 34.0 | 27.1 | 118.5 | 121.9 |
| PA Pulse Pressure | 17.6 | 14.5 | 38.9 | 55.4 |
| Cardiac Output | 4.4 | 5.1 | 5.5 | 4.9 |

Hemodynamic measurements of vorinostat effects in calves with hypoxia-induced pulmonary hypertension. Data were obtained from right heart catheterization as described in Materials and Methods. Standard units: Pressure, mm Hg; Cardiac output, L/min; PA, pulmonary artery.

Figure Legends

Figure 1. Apical 4-chamber echo images. Apical 4-chamber end-diastolic echo recordings were obtained from the study calves as described in Methods. Corresponding animals are shown to Figs. 1 and 2. Equivalent images were obtained at end-diastole for each animal. Arrow indicates septal distortion in PH non-treated calf, which is not present in PH vorinostat-treated calf.

Figure 2. Comparison of inflammatory and matrikine gene expression in vorinostat treated versus untreated hypoxic calves. mRNA abundance was quantitated as described in Methods. For each indicated mRNA, abundance in the vorinostat-treated PH calf was determined relative to that in the untreated PH calf (indicated by dotted line at unity).
